# Examination of the Effect of Suitable Size of Shoes under the Second Metatarsal Head and Width of Shoes under the Fifth Metatarsal Head for the Prevention of Callus Formation in Healthy Young Women

**DOI:** 10.3390/s18103269

**Published:** 2018-09-28

**Authors:** Ryutaro Kase, Ayumi Amemiya, Rena Okonogi, Hiroki Yamakawa, Hisayoshi Sugawara, Yuji L. Tanaka, Masatoshi Komiyama, Taketoshi Mori

**Affiliations:** 1 Department of Nursing Physiology, Graduate School of Nursing, Chiba University, 1-8-1 Inohana, Chuo-ku, Chiba-shi, Chiba 260-8672, Japan; rkase@chiba-u.jp (R.K.); h.sugawara@chiba-u.jp (H.S.); yuji@faculty.chiba-u.jp (Y.L.T.); mkomi@faculty.chiba-u.jp (M.K.); 2 Department of Nursing, Hospital of Jichi Medical University Saitama Medical Center, 1-847 Amanuma-cho, Oomiya-ku, Saitama-shi, Saitama 330-8503, Japan; leana-viking2013@ezweb.ne.jp; 3 Nature’s Walk Ltd., 3-2-3 Honcyou, Chuo-ku, Chiba-shi, Chiba 260-0012, Japan; yamakawa@natureswalk.co.jp; 4 Department of Life Support Technology (Molten), Graduate School of Medicine, The University of Tokyo, 7-3-1 Hongo, Bunkyo-ku, Tokyo 113-0033, Japan; tmoriics-tky@umin.ac.jp

**Keywords:** shear stress, shoe, callus, walking, woman

## Abstract

Excessive pressure and shear stress while walking cause a risk of callus formation, which eventually causes foot ulcers in patients with diabetes mellitus. Callus under the second metatarsal head (MTH) has been associated with increased shear stress/pressure ratios (SPR). Callus under the fifth MTH has been associated with increased peak shear stress (PSS). The purpose of this study is to examine whether the effect of the suitable size and width of shoes prevents diabetic foot ulcers under the second and fifth MTH. We measured the pressure and shear stress by testing three kinds of sizes and two types of width of shoes. Significant difference was not observed in the SPR under the second MTH among different sizes of shoes. However, the pressure and shear stress were significantly lower when putting on shoes of fit size compared with larger sizes. The PSS under the fifth MTH was significantly smaller when putting on shoes of fit width compared with those of narrow width. Wearing shoes of fit size and width has the potential to prevent callus formation by reducing the pressure and shear stress constituting SPR under the second MTH and PSS under the fifth MTH.

## 1. Introduction

From 1980 to 2014, the number of patients with diabetes mellitus has increased from 108 million to 422 million [1]. The global prevalence of diabetes among adults over 18 years has also increased from 4.7% to 8.5% in the same period [1]. According to a projection by the World Health Organization (WHO), diabetes will be the seventh leading cause of mortality by 2030 [2]. Diabetes is associated with several complications, including neuropathy, retinopathy, and diabetic kidney disease, of which diabetic foot ulcer constitutes one that develops in 4–10% of patients with diabetes [3]. Reportedly, 25% of patients with diabetes experience diabetic foot ulcers within their lifetime [3]. Moreover, diabetic foot ulcers show frequent recurrence, and 7–20% of patients with diabetic foot ulcers have to undergo leg amputations, accounting for a 15–40 times higher ratio of leg amputation in patients with diabetes compared with healthy people [4]. Foot ulcers not only lead to amputation of the lower limb, they also adversely affect individuals’ quality of life (QOL), reduce physical activity, and aggravate psychological stress [5]. A study has estimated that diabetic ulcer-related costs averaged over $13,000 per episode, excluding costs associated with psychosocial issues, decline in the QOL, and lost productivity [6]. Thus, the prevention of diabetic foot ulcers is imperative not only for patients, but also both socially and economically.

Callus is one of the leading causes of diabetic foot ulcers. Callus occurs by the thickening of the stratum corneum by repeated pressure and overloading [7]. Reportedly, callus formation precedes ulcer formation in over 82% of patients with diabetic foot ulcers [7]. In addition, the relative risk for ulceration in a callused region is 11.0 compared with that without callus [8,9]. Therefore, the prevention of callus leads to the prevention of diabetic foot ulcers.

It has been reported that 42% of lower limb amputations are estimated to be shoe-related, 8% of which could have been avoided by wearing appropriate footwear [10]. A report has mentioned that the pressure on the plantar during walking is not significantly different between the patient group using a custom-made insole and that using prefabbed insoles [11], suggesting that changing merely the insole has no effect on reducing the pressure on the plantar. Presently, custom footwear with confirmed efficacy in preventing callus formation is available [12,13]. However, in the clinical setting, it has been reported that only 22% patients with diabetes have worn custom footwear [14]. This is possible because patients with diabetes do not recognize the importance of callus formation prevention, and custom footwear is expensive as well. In addition, it is not feasible to wear customized footwear to merely prevent callus formation. In this study, it was hypothesized that pressure and shear stress decreased if prefabric footwear was suitable in width and size, which is effective in preventing callus formation.

Several studies have measured the pressure on the plantar of patients with diabetes during walking. Repeated pressure and shear stress during walking contribute to callus formation on the plantar region [15,16]. In fact, patients with callus, even after removing the callus, exhibit significantly higher peak pressure (PP) during walking than patients without callus [17]. A report has stated that both plantar shear stress and shear stress–time integral values are elevated in diabetic patients with peripheral neuropathy, suggesting the potential clinical significance of these factors in ulceration [18]. Despite the potential significance of shear stress, few researches have measured shear stress because of technical difficulty, especially during walking.

However, ShokacChip^TM^ (Touchence Inc., Tokyo, Japan), a newly developed sensor, has facilitated the measurement of pressure and shear stress applied to an insole of footwear. Since the measurement method was new, this study was measured for young healthy subjects. Using ShokacChip^TM^, a study has revealed that callus is associated with an increased shear stress/normal stress (pressure) ratios (SPR) under the second metatarsal head (MTH) and with higher peak shear stress (PSS) under the fifth MTH [19]. In addition, it has been suggested that callus formation under the second MTH is caused by wearing large shoes, and that callus formation under the fifth MTH is associated with wearing narrow shoes [20]. Thus, it was hypothesized that wearing shoes of suitable size and width is effective to reduce pressure and shear stress under the second and fifth MTH. In other words, it was hypothesized that it leads to the prevention of callus formation. To the best of our knowledge, no study has yet investigated differences in the pressure and shear stress among various sizes and widths of shoes.

Thus, the present study aimed to examine the pressure and shear stress under the second and fifth MTH during walking and confirm whether there is a difference between suitable shoes and large shoes that can lead to the prevention of callus formation. In addition, the present study aimed to confirm whether there is a difference between suitable shoes and narrow shoes that can lead to the prevention of callus formation.

## 2. Materials and Methods

### 2.1. Research Design and Procedure

In this study, variables were compared with crossover design. This study was conducted and reported according to a part of a Consolidated Standards of Reporting Trials (CONSORT) checklist [21].

### 2.2. Participants Recruitment and Ethics

In this study, 49 healthy adults without diabetes who could walk without support were enrolled as study participants using a snowball sampling method. This study focused on women, since they tended to wear incorrectly sized shoes [22,23]. This study was conducted in accordance with the Declaration of Helsinki, and the protocol was approved by the Ethical Committee of the Graduate School of Nursing, Chiba University (28-65: November/22/2016) (Chiba, Japan). In addition, written informed consent was obtained from each participant before enrollment.

### 2.3. Footwear Type

In this study, a skilled prosthetist and orthotist measured participants’ feet sizes and selected a pair of fit shoes for each participant. The experimental shoes that were used prepared different sizes of the same product. These shoes were made from leather and were brand shoes called gute wahl. These shoes were recommended by Shoes Meister for diabetic foot ulcers. Three sizes of experimental shoes—fit size, 1-cm larger size and 2-cm larger size—were tested to measure the pressure and shear stress under the second MTH. For the fifth MTH, the following two types of experimental shoes were tested: fit width and narrow width. Experimental shoes of narrow width were prepared by inserting a cork insole of 6-mm thickness in the fit shoes. The cork insole was prepared by the prosthetist and an orthotist by cutting a cork sheet. The cork insole was thinned only at the heel for preventing the shoes from coming off during walking.

### 2.4. Pressure and Shear Stress under the Second MTH

Participants put on unified socks during the test. If there was a callus, at certain locations on the plantar surface, eventually sensors read higher values as calluses increase the tissue rigidity. In the case of calluses on the plantar region, all of the calluses were removed prior to data collection in this study. First, the prosthetist and orthotist obtained participants’ footprints. Next, the foot length and width of standing participants were measured, and a fit size of shoes was selected. Second, the researcher directly attached sensors of ShokacChip^TM^ (Touchence Inc.) on the plantar region of participants. Then, converters were put on the front shin portion and wirelessly connected to a personal computer. All of the participants tested the three sizes of shoes. The prosthetist and orthotist assisted participants with putting on the shoes in order to avoid any difference in the manner of shoe wearing. The order of testing the three sizes of shoes was randomly selected. Third, participants walked approximately 15 m as practice for confirming that they did not experience any pain or interference on walking with the attached sensors. Lastly, external force (pressure and shear stress) was measured (details in “Data Collection”). All of the participants walked approximately 15 m twice as the measurement walk, and the researcher recorded all of the sensor data, ensuring that the sensors were operating during the study. In addition, an assistant measured 15 m of walking time using a stopwatch. After obtaining the measurements for one size of shoes, measurements were obtained for the other two types of shoes in the same manner.

### 2.5. Measurement of Pressure and Shear Stress under the Fifth MTH

Measurements were obtained for the fifth MTH via the same procedure that was used for the second MTH. However, in this case, only two types of shoes, fit width and narrower than fit width, were tested.

### 2.6. Data Collection

The pressure and shear stress were measured using ShokacChip^TM^. The high sensitivity was realized for three-dimensional axes by processing three piezoelectric elements and locating them at three-dimensional axes on the 2-mm^2^ chip. Notably, the sensor size was φ10.0 × 1.3 mm (t); as it was very small and thin, it could measure the in-shoe pressure and shear stress of the plantar under each MTH region. The reliability and validity of the system for measuring the in-shoe plantar pressure and shear stress have been previously established [24]. In this study, coefficients of variation (CV) and intraclass correlation coefficients (ICC) were confirmed. The mean CV was 9.7%. The mean ICC was 0.943 [24]. Even though this sensor does not require any calibration before each measurement, calibration was performed before measuring with shoes.

In this study, the external force variables, including PP, pressure time integral (PI), PSS, and shear stress integral (SSI) of each walking cycle were calculated from the data of external force recorded by the sensor. These variables have been frequently used in previous studies [18,19,20,24]. In addition, the SPR, which is considered to be associated with callus formation, was calculated by dividing the shear stress by the normal stress (pressure), concretely, using peak value (SPR-p) and time-integral value (SPR-i, calculated by dividing the SSI by the PI value). Furthermore, data regarding age, sex, height, weight, body mass index (BMI), the number of callus under the second MTH and fifth MTH, foot length, and foot width were obtained.

### 2.7. Data Analysis

The variables of the external force were obtained by averaging 12 steps after removing the first and last three steps. Data analysis was performed for each second and fifth MTH by using MATLAB R2012a (The Math Works, Inc., Natick, MA, USA). Statistical analyses were performed using IBM SPSS Statistics ver.23.0 (Chicago, IL, USA). The Cohen’s d effect size was 0.45, and sample size was 41 when calculated based on the previous study [25]. Normality analyses were conducted before data analysis. A paired *t*-test was conducted to compare the external force value due to shoe differences. In this study, p = 0.05 two-tailed was considered statistically significant. In addition, the calculated Cohen’s d effect size was described in the results. Descriptive data were expressed as the mean ± standard deviation of continuous variables and n (%) for categorical variables. The external force values were represented as bar graphs in these figures. These bar graphs in the figure contained standard errors. Further, Pearson’s test was used whether there was a correlation between the difference of width (shoes versus feet) and the PSS.

## 3. Results

In this study, 49 participants were enrolled, and 54 and 44 feet were used for obtaining measurements of variables for the second and fifth MTH, respectively. Table 1 summarizes the characteristics of all of the participants. Calluses under the second MTH were observed in 29 of 54 feet, whereas those under the fifth MTH were observed in nine of 44 feet.

**Figure 1:**
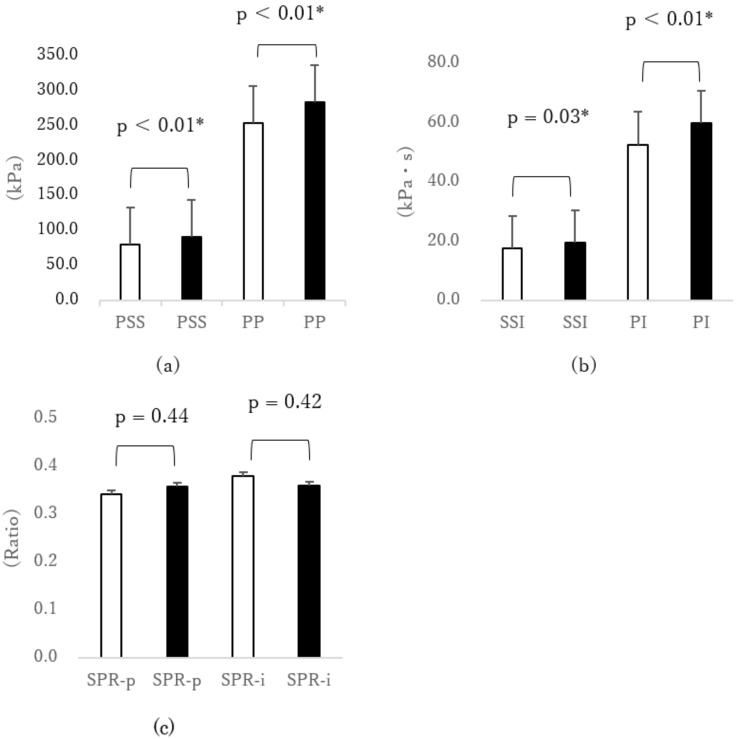
Comparison of external force under the second MTH due to differences in shoe size (□ fit size vs. ■ 1-cm larger size). (**a**) Peak normal stress (pressure) (PP) and peak shear stress (PSS), (**b**) normal stress (pressure) time integral (PI) and shear stress time integral (SSI), and (**c**) shear stress/normal stress (pressure) ratio of peak value (SPR-p) and shear stress/normal stress (pressure) ratio of time integral value (SPR-i). * p < 0.05 paired *t*-test. Error bar was standard error (SEM). MTH: metatarsal head.

**Figure 2:**
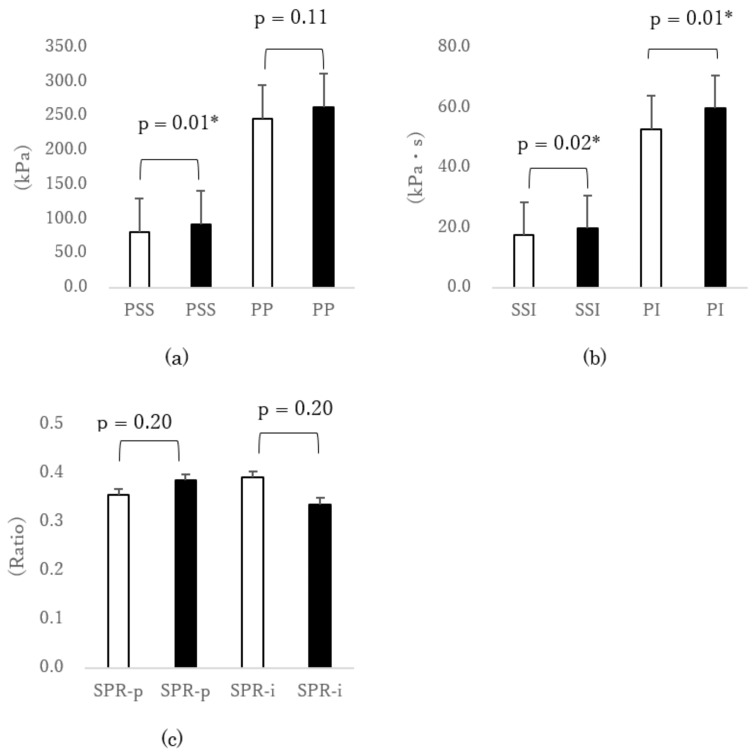
Comparison of external force under the second MTH due to differences in shoe size (□ fit size vs ■ 2-cm larger size). (**a**) Peak normal stress (pressure) (PP) and peak shear stress (PSS), (**b**) normal stress (pressure) time integral (PI) and shear stress time integral (SSI), and (**c**) shear stress/normal stress (pressure) ratio of peak value (SPR-p) and shear stress/normal stress (pressure) ratio of time integral value (SPR-i). * p < 0.05 paired *t*-test. Error bar was standard error (SEM). MTH: metatarsal head.

When putting on shoes of fit size, the PSS under the second MTH was significantly smaller than those putting on larger sizes [1-cm large size: p < 0.001, d = 0.40 (Figure 1a); 2-cm large size: p = 0.01, d = 0.53 (Figure 2a)]. In addition, the PP under the second MTH was significantly smaller (p = 0.001, d = 0.33) when testing shoes of fit size compared with shoes of 1-cm large size (Figure 1a). The PI under the second MTH in cases of fit-sized shoes was significantly smaller than those in cases of larger sizes [1-cm large size: p = 0.002, d = 0.36 (Figure 1b); 2-cm large size: p = 0.012, d = 0.35 (Figure 2b)]. In addition, the SSI under the second MTH was significantly smaller (p = 0.034, d = 0.28) when putting on the fit size than when putting on the 1-cm large size (Figure 1b). Furthermore, the SSI under the second MTH was significantly smaller (p = 0.023, d = 0.38) when testing the fit size than when testing the 2-cm large size (Figure 2b). Thus, the SSI significantly increased when participants put on larger shoes. However, even when different sizes of shoes were tested, significant difference was not observed in the SPR-p and SPR-i, which were associated with the callus formation under the second MTH.

**Figure 3:**
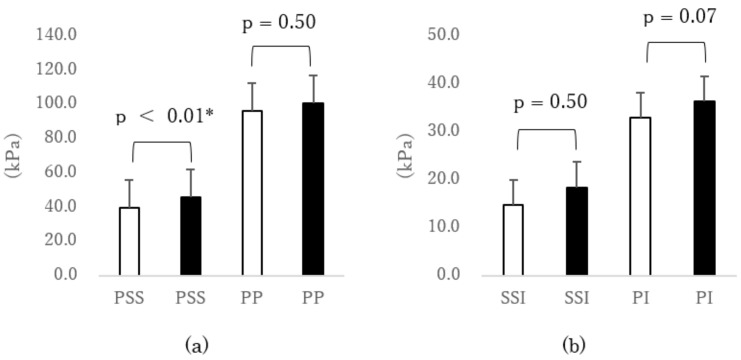

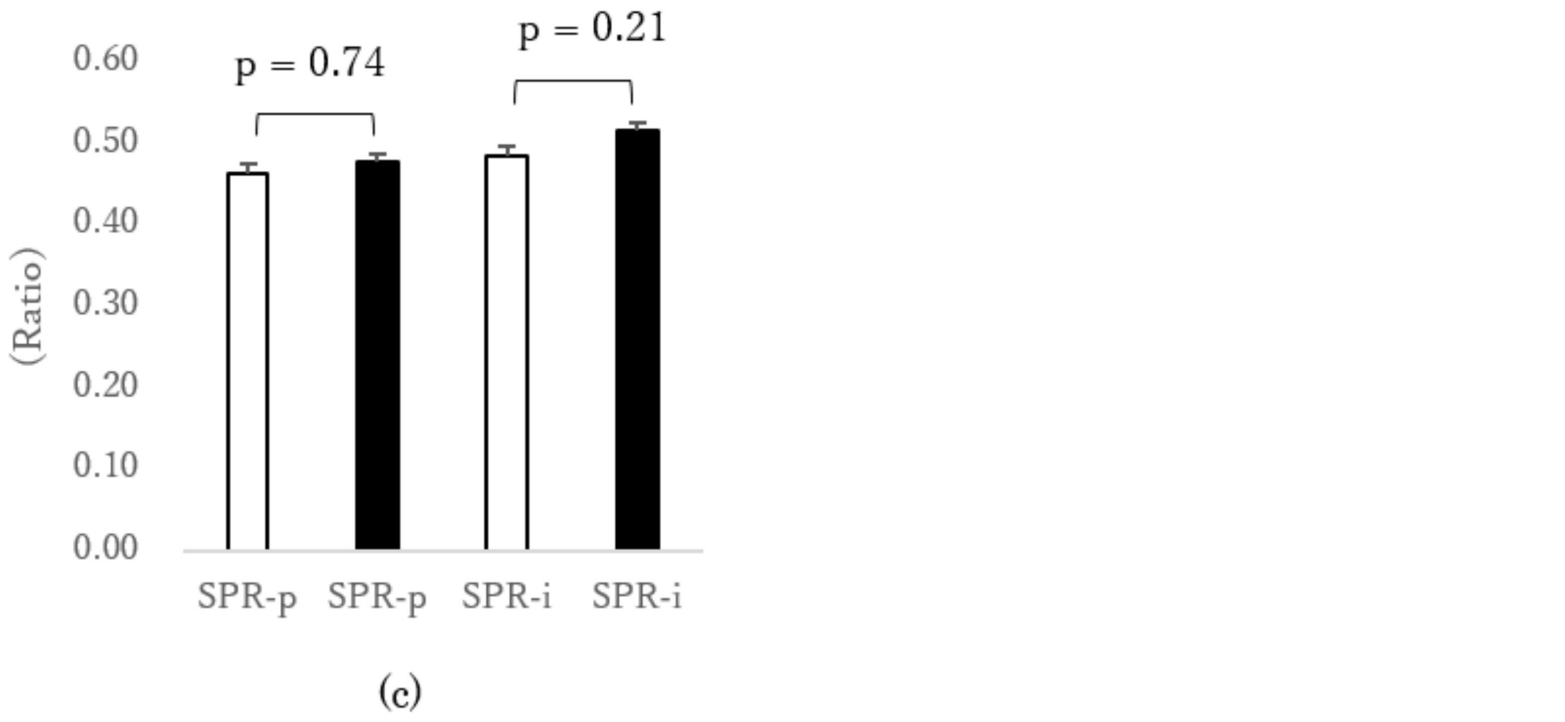
Comparison of external force under the fifth MTH due to differences in shoe width (□ fit width vs. ■ narrow width). (**a**) Peak normal stress (pressure) (PP) and peak shear stress (PSS), (**b**) normal stress (pressure) time integral (PI) and shear stress time integral (SSI), and (**c**) shear stress/normal stress (pressure) ratio of peak value (SPR-p) and shear stress/normal stress (pressure) ratio of time integral value (SPR-i). * p < 0.05 paired *t*-test. Error bar was standard error (SEM). MTH: metatarsal head.

The PSS under the fifth MTH was significantly smaller (p < 0.01, d = 0.42) when putting on shoes of fit width than when putting on shoes of narrower width (Figure 3a). However, significant differences were not observed in the others under the fifth MTH.

**Figure 4:**
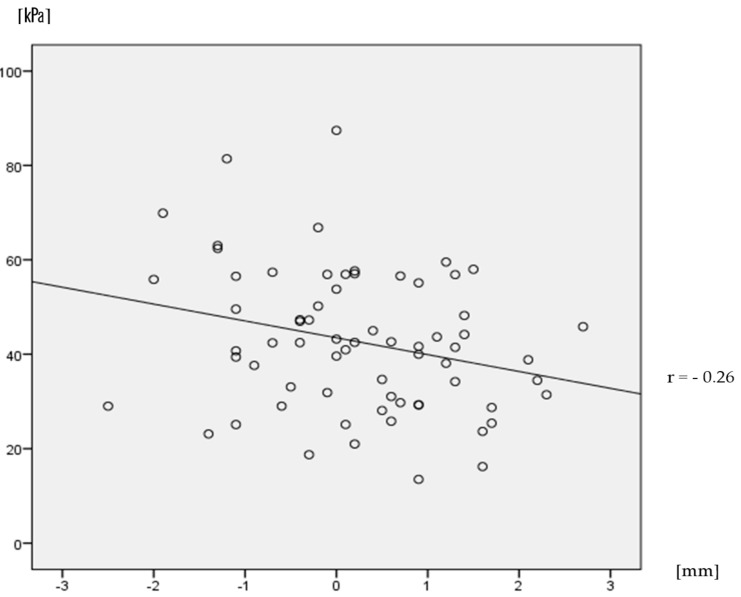
Relationship between the difference of width (shoes vs. feet) and PSS. The straight line is the correlation line. Dots are samples.

The difference between the participants’ foot width and shoe width was seven mm on average in case of shoes of fit width, in which shoes were wider than feet. In contrast, the average of the difference was −3 mm for narrow shoes, in which shoes were narrower than feet. The correlation coefficient between the difference of width (shoes versus feet) and the PSS was −0.26, which was only a weak negative correlation (Figure 4).

## 4. Discussion

This is the first study to reveal the pressure and shear stress under the second and fifth MTH when young healthy participants put on shoes of different sizes or widths for preventing callus formation. For the second MTH, the findings of this study revealed that the PI and SSI decreased by wearing suitably sized footwear compared with larger sized footwear; these valuables comprised the SPR that is associated with the prevention of the callus formation. In addition, the findings also revealed that the PSS associated with the callus formation at the fifth MTH decreased by wearing footwear of suitable width.

Regarding the shoe size, the PI, SSI, and PSS under the second MTH were significantly smaller when putting on fit-sized shoes compared to larger-sized shoes. The PI and SSI comprise SPR, which is associated with the prevention of callus formation [19]. However, significant difference was not observed in the SPR-i that is associated with the callus formation under the second MTH, because in this study, both SSI and PI decreased by putting on fit shoes. Assumedly, as the large sliding of the plantar occurs when walking, wearing larger-size shoes results in an increase in the PI, SSI, and PSS under the second MTH. Thus, it has been clear that unsuitable shoes augment pressure and shear stress. Wearing suitably sized shoes is effective at reducing pressure and shear stress and leading to the prevention of callus formation under the second MTH.

Regarding the width of shoes, the PSS under the fifth MTH was smaller when putting on shoes of fit width compared with those of a narrow width. In addition, the correlation coefficient between the difference between the foot width and shoes width and the PSS was −0.26, which was a weak negative correlation in the range between narrow shoes and shoes that fit (Figure 4). The results of this study support that the width of the outdoor shoes was narrow, which is related to the callus formation [26]. The relative risk for callus formation is 2.03 when comparing wearing narrow shoes with suitable shoes, and the relative risk for corn formation is 6.18, respectively [26]. Further, callus formation has been shown to be associated with higher PSS under the fifth MTH [19]. The present data proved a previous suggestion that callus formation under the fifth MTH is associated with wearing narrow shoes [20]. As the fifth MTH has a thinner subcutaneous tissue than the first and second MTH regions, the mechanical load may not be absorbed by the subcutaneous tissue. From the results of this study, it is considered that if a person wears narrow shoes, the shear stress increases, because the foot is pressed against both sides of the shoes. On the other hand, when wearing fit shoes that are somewhat wider than feet, the foot spread by the load is not pressed against the sides of the shoes, and the shear stress is decreased. Thus, it is suggested that wearing footwear of suitable width has been effective at reducing pressure and shear stress, and thus preventing callus formation under the fifth MTH.

A previous study on shoe selection has reported that the use of incorrectly sized shoes significantly correlates with pain and ulceration [27]. Another study that is based on data from 227 women has reported that 48.5% were wearing incorrectly sized shoes, of which 12.8% were wearing shoes that were at least 1 cm larger than their feet [23]. A study that examined 65 older adults verified that 72% participants were, in fact, wearing incorrectly sized shoes (65% were wearing shoes larger than their feet) [27]. On the contrary, a study evaluating 356 women (age 20–60 years) has reported that 88% of patients were wearing shoes that were narrower than their feet [22]. As the proportion of older adults is high among patients with diabetes [28], they are more likely to wear larger-size shoes. Moreover, it has been revealed that an incorrect width of shoes has been worn by people who selected incorrect shoes [23]. Therefore, it is possible that patients with diabetes tend to frequently wear unsuitable shoes. Perhaps, it is difficult for people to select suitable shoes because it is difficult to find shoes with all applicable sizes, widths, and shoe fittings in everyday life. This might be why patients with diabetes frequently wear unsuitable shoes. It would very be important for patients with diabetes to find suitable shoes at least regarding the points of size and width in order to reduce the pressure and shear stress that lead to callus formation.

This study has some limitations. First, all of the participants were healthy people without diabetes. Compared with healthy participants, patients with diabetes or diabetic neuropathy have abnormal plantar pressure distribution as follows: elevated plantar pressure because of the motion of small rolling during the mid-stance [29], an impaired ability to stabilize their body when walking on irregular surfaces [30], foot deformity [31], limitation of the joint range of motion, and muscle weakness [32,33]. Second, patients with diabetes are often older than the participants of this study. Thus, it is quite likely that these characteristics of patients with diabetes affect the results in the clinical setting. In future, it is necessary to assess the effect of wearing shoes of suitable size and width in patients with diabetes. Third, since only women were included in this study, the findings of this study are limited to woman. Finally, an insufficient sample size due to a lack of shoe size has affected the results. Data collection was finished before reaching the target sample size, since the shoe size could not have been sufficiently collected, and data collection time was limited. In future study, it will be important to increase the sample size and include males, a diabetic cohort, and a higher body mass index cohort.

## 5. Conclusions

This is the first study to compare the pressure and shear stress under the second and fifth MTH from wearing different sizes of shoe in young healthy participants. Apparently, the PI and SSI under the second MTH decrease by wearing suitably sized shoes compared with larger shoes. Furthermore, the PSS under the fifth MTH decreases by wearing shoes of a suitable width rather than narrow shoes. Therefore, there is a possibility that wearing shoes of suitable width and size can reduce pressure and shear stress and thus lead to the prevention of callus formation and diabetic foot ulcers.

## Figures and Tables

**Table 1:** Patient characteristics.

	Measurement of Second MTH	Measurement of Fifth MTH
Number of patients	27	22
Number of feet	54	44
Age (y)	26.9 ± 7.2	26.7 ± 7.3
Sex (n)		
Man	0	0
Woman	27 (55%)	22 (45%)
Height (cm)	156.0 ± 5.0	157.2 ± 4.6
Weight (kg)	56.9 ± 10.0	55.4 ± 9.3
BMI	23.1 ± 3.7	22.4 ± 3.5
Number of feet with callus under the second MTH	29 (53.7%)	-
Number of feet with callus under the fifth MTH	-	9 (20.5%)
Feet length (cm)	23.0 ± 0.9	22.9 ± 1.0
Feet width (cm)	22.1 ± 2.0	22.6 ± 1.3

n (%); mean ± SD, BMI; body mass index, MTH; metatarsal head.
